# Investigating the Immediate Influence of Moderate Pedal Exercises during an Assembly Work on Performance and Workload in Healthy Men

**DOI:** 10.3390/healthcare9121644

**Published:** 2021-11-27

**Authors:** Mohammed H. Alhaag, Atef M. Ghaleb, Lamjed Mansour, Mohamed Z. Ramadan

**Affiliations:** 1 Industrial Engineering Department, King Saud University, Riyadh 11421, Saudi Arabia; amag16@gmail.com (A.M.G.); mramadan1@ksu.edu.sa (M.Z.R.); 2 Zoology Department, College of Science, King Saud University, Riyadh 11421, Saudi Arabia; lmansour@ksu.edu.sa

**Keywords:** cognitive task, EEG, ECG, exercise, sedentary behaviors, occupational safety

## Abstract

Physical inactivity has increased in prevalence among adults in industrialized and developing countries owing to the fact that the majority of job situations require individuals to remain seated for extended periods of time. This research aims to evaluate the influence of cycling on a stationary bike while executing a keyboard assembly task on the task completion time, error percentage, and physiological and subjective measurements. The physiological measures were electroencephalography (EEG) and electrocardiographic (ECG) signal responses, whereas the subjective measures were subjective workload ratings and subjective body discomforts. Two variables were evaluated, namely assembly methods (with versus without pedal exercises at a moderate intensity) and session testing (pre- versus post-test). Thus, the repeated measures design (i.e., assembly method by session testing of participants) was used. According to the completion time, error %, participant self-reports, and ECG and EEG statistical analysis data, the participants’ performances in the keyboard assembly task did not decrease while they performed pedaling exercises (*p* > 0.05). Additionally, when participants completed the assembly task while executing the pedaling exercises, the mean inter-beat (RR) intervals significantly reduced (*p* < 0.05) while the mean heart rate increased (*p* < 0.05), which mean that pedaling exercises caused physical workloads on the participants. Participant performance was unaffected by performing a workout while performing the assembly activity. Thus, administrations should encourage their employees to engage in short sessions of moderate-intensity exercise similar to the suggested exercise in the study to improve a person’s physical health during work without interfering with the effectiveness of work.

## 1. Introduction

Physical inactivity has become more prevalent in working environments in developed and developing countries because in most work environments employees are required to remain seated for extended periods of time [1]. Scientists have provided the community with several modern devices for transportation, communication systems, and home entertainment that have become principal parts of our daily lives, thus changing the social and physical environments in which we currently live [2]. Therefore, the demand for the present working population to be physically active has declined significantly [3,4]. Sedentary behaviors are characterized by long periods of sitting or the absence of physical activity, as evidenced by their association with low-energy expenditure values [2,3]. These behaviors may lead to adverse biological consequences, such as increased cardiovascular risk and body weight gain [5,6,7,8,9,10,11,12].

Strong evidence has shown that the risks of many adverse health conditions, including major non-communicable diseases such as coronary artery disease and other related diseases of older adults, may increase owing to physical inactivity [13,14,15,16,17]. In addition, sedentary behavior has been linked to poor health outcomes in adults, including all-cause mortality, cardiovascular disease mortality, and cancer mortality, as well as the incidence of cardiovascular disease, type 2 diabetes, and cancer [18,19]. Recently, physical inactivity has been recognized as the fourth leading risk factor for global mortality and accounts for 6% of all deaths [20]. In contrast, moderate to vigorous physical activities (MVPAs) have been suggested to reduce the risk of cardiovascular, regulate type 2 diabetes, avoid the occurrence of certain kinds of cancers as well as increase the density of bone, improve psychological health, and decrease overall mortality [18,20,21,22]. According to the World Health Organization, all healthy adults (i.e., ≥18–64 years) should be engaged in moderate-intensity physical activity for at least 30 min on 5 separate days of the week or vigorous-intensity physical activity for at least 20 min on 3 separate days of the week [18]. As a result, healthcare researchers have stressed the value of physical activity and the health advantages associated with low-intensity exercise [23,24,25]. The occupational health hazard domain can be a key area for both the prevention of health conditions associated with physical inactivity and the appropriate intervention in work environments [5]. Hence, ergonomic policies regard the execution of physical workload as an opportunity to reduce sedentary behaviors at work as a priority, emphasizing sitting as the prime outcome for future workplace action [8,26].

The uses of treadmill and cycling desks, as well as micro-breaks, have been recommended as strategies to reduce the time spent sitting at work [27,28]. Carr et al. [29,30] investigated the practicality and feasibility of a portable pedaling exercise equipment on middle-aged participants, primarily female, who worked in sedentary environments and randomized them into an intervention and a controlled group. The results showed that biking at a moderate speed was able to replace about one hour of daily sedentary time. In a similar study, Rovniak et al. [31] indicated that the introduction of pedaling devices can help expend nearly 90 kcal/h extra compared with the number of calories that would have been expended by sitting. Although such interventions have demonstrated encouraging results even in the short term [27], poor methodology quality [26], investment costs [32], and concerns regarding staff productivity [24] have limited the implementation of large-scale interventions in the workplace.

Electroencephalography (EEG) is a technique for recording the brain’s electrical activity. Numerous studies have demonstrated that a fluctuation in EEG rhythm can accurately predict poor performance due to changes in mental activities [33,34]. Other research has investigated variations in EEG waves during the performance of continuous and challenging activities, with the most notable occurrence being an increase in theta strength of the frontal cortex EEG. This increase has been reported in tasks involving visual search [35], viewing a 3D display [36], and loading of working memory. Additionally, it has been found that the alpha power of the EEG decreases during demanding and cognitive tasks. This decrease occurred in the frontocentral and parietal regions [37,38]. Zhao et al. [39] demonstrated a large rise in the alpha and theta powers of the EEG and a significant decrease in the beta power in several scalp regions. Additionally, they noticed a drop in beta power in the frontal areas.

The electrocardiographic (ECG) signal is a graphic recording of the electrical activity of the heart during the cardiac cycle. A general characteristic of the ECG signal is heart rate variability (HRV). HRV is another technique that is often understood as a physiological response to the cardiovascular system’s reaction to mental stresses [40,41]. HRV has become a growing interest because it is an indicator of cardiovascular autonomic function, and cardiovascular risks and all-cause mortality are directly predicted by reducing HRV [42,43]. In addition, reduced coronary flow reserves and antedate episodes of dynamic myocardial ischemia are associated with parasympathetic withdrawal quantitated by HRV [44]. HRV is a term that refers to periodic fluctuations in heart rate (HR) and serves as an indicator of the autonomic nervous system’s activity level [45]. 

Among the HRV indices, the standard deviation of normal RR intervals (SDNN) has been suggested to reflect global variability, whereas the root mean square of successive RR interval differences (RMSSD) and high-frequency (HF = 0.15–0.4 Hz) power have been associated with vagal activity [46]. Additionally, the ratio of the absolute power in low-frequency bands (LF = 0.04–0.15 Hz) to the absolute power in high-frequency bands (LF/HF) is interpreted as a measure of the sympathetic–parasympathetic balance [47]. Hallman et al. [48] revealed that physical inactivity is associated with decreased HRV, particularly regarding HF power, thus reflecting the reduced cardiovascular autonomic control. Moreover, inactivity caused by extended bed rest reduces HRV [49]. Previous studies have reported that sedentary time results in alterations of cardiovascular health and HRV during prolonged bed rest [50,51]. In addition, declining resting HRV in adults, especially with regard to parasympathetic HRV measures, is linked with insufficient moderate-to-vigorous physical activities [52,53].

This work presents an effort to reduce the adverse effects of prolonged sitting during the execution of an assembly task while considering the prospective benefit to both health and work performance. The objective of this research was to evaluate the influences of cycling on a stationary bike while executing an assembly task on human performance based on the completion task time, error percentage, and physiological and subjective measurements. The research question was: would the performance of the participants in executing assembly tasks be affected if they performed pedaling exercises while executing the assembly tasks? The effect of introducing the bicycle pedaling exercise on human workload and performance during the execution of an assembly activity was investigated using a variety of measurements. The physiological measures were electroencephalography (EEG) and ECG signal responses, whereas subjective measures were subjective workload ratings and subjective body discomforts. The main contribution of this study was the use of EEG and HRV parameters while the participant performed a bicycle pedaling exercise and a cognitive task. The cognitively demanding tasks in our study included visual search, compare, decision making, memory, attention, judgment, and inserting keys in appropriate locations. Therefore, including physical work (i.e., exercise) to that cognitive task could affect the participants’ performances. ECG and EEG signals were introduced as response variables, which were very relevant for both tasks.

## 2. Materials and Methods

### 2.1. Participants

Sixteen healthy male graduate students from King Saud University volunteered in this study. The same procedure for participant recruitment by Ramadan and Alhaag [36] and Ramadan [54] was employed in this study. Table 1 presents the mean and standard deviation of the participants’ characteristics. Effect size was computed by eta-squared (ƞ^2^), and deemed as: without effect if 0 < ƞ^2^ ≤ 0.04; minimum if 0.04 < ƞ^2^ ≤ 0.25; moderate if 0.25 < ƞ^2^ ≤ 0.64; and strong if ƞ^2^ > 0.64. To calculate sample power, the following assumptions were made:

- An expected medium/moderate effect size (for example, f = 0.25);
- A 5% probability of error for 95% power;
- Two groups (i.e., assembly task with cycling and assembly task without cycling);
- A correlation among both repeated measures of 0.5;
- A correction for nonsphericity of 1.

These inputs resulted in a sample size of at least 15 participants at each level, which was less than the experiment’s recruited participants. The number of participants was also in line with other studies with a similar topic and a comparable laboratory set-up; for example, Kruse et al. [55] used 13 subjects, Kowalsky et al. [56] used 14 subjects, and Perdomo et al. [57] utilized 15 participants. 

Each participant was proficient at performing computer tasks, and none disclosed having a musculoskeletal disorder. We recruited healthy but physically inactive students (self-reporting no physical activity in their daily life). Participants were also not recreationally active in sports and were unfamiliar with cycling exercise. Participants were instructed to have a full night’s sleep and abstain from smoking and caffeine for eight hours before testing. Prior to conducting the tests, participants signed an informed consent form approved by the Institutional Review Boards of King Saud University and the College of Medicine (E-194247, 28 November 2019).

### 2.2. Experimental Design

In this study, two variables were evaluated; these were assembly methods (i.e., executing an assembly task while cycling versus performing the same task without pedal exercises) and session testing (pre- versus post-test). Thus, the repeated measures design (i.e., assembly methods by session testing of participants) was implemented as a representation of the experiment. During the pre-and post-test sessions, the EEG and ECG signals were recorded for 5 min each to reduce the noise signals, such as hand and leg movement. EEG and HRV indices, task completion time, error percentage, subjective workload assessments (NASA TLX), and whole-body discomfort scores were used as dependent variables. 

### 2.3. Task

**Figure 1:**
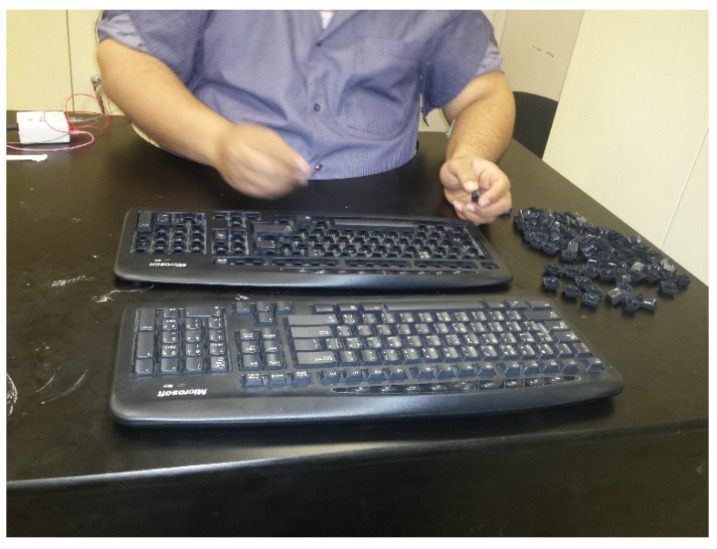
The assembly task of the study.

The selected task for this study was the assembly of computer keyboards. The participants were positioned on a stationary bike to assemble a keyboard consisting of 103 keys and 13 pins. Two keyboards were provided to each participant; one keyboard would be assembled by the participant and the other served as the guide for the assembly operation, as shown in Figure 1. The participants performed the experimental trial on an ergometer bike while assembling a keyboard and performing this with and without pedaling.

### 2.4. Experimental Setup and Procedures 

**Figure 2:**
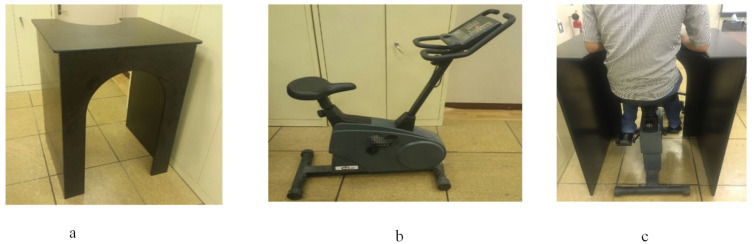
Experimental setup; (**a**) an office workstation; (**b**) stationary bike; (**c**) adjusting work station.

The experimental procedures were initially established by defining the experimental and environmental conditions. The office workstation with dimensions of 0.92 m (L) × 0.82 m (W) × 1.13 m (H) that was created in an ergonomics laboratory for this study is shown in Figure 2a; the average temperature and relative humidity were 24 °C and 29.8%, respectively. The ergonomic bike bicycle ergometer, as shown in Figure 2b, was used in this study, and was calibrated to the intensity level of 2 at 50 revolutions per minute (cycling at a moderate intensity of 60 Watt). The participants were instructed to ride on the bicycle on the preferred seating level and they maintained the upper body in an upright orientation where they could adjust the height of the seat on their desktop. The height and depth of the desk were fixed for the participant throughout the tests, as shown in Figure 2c.

The procedure for this study required that participants would complete a task under specific experimental settings, namely assembling a keyboard while conducting pedaling exercises at a pace of 50 revolutions per minute and with light power output and without performing the exercises. A study by Tiwari et al. [58] recommended that, for daylong cycling work, the power output should be limited to 60 W (light power output) and the cycling rate should be 50 revolutions per minute. They argued that the participants’ physiological responses were within acceptable limits for continuous cycling work.

The participants were provided with the description and introduction to the simulated office workstation and the pedaling exercise. Anthropometric measures such as stature height, elbow height, and elbow sitting height were obtained, and each participant filled out a demographics questionnaire. Then, the participants were instructed on how to correctly use the stationary bike, with the option of adjusting the bike seat height to their comfort level.

**Figure 3:**
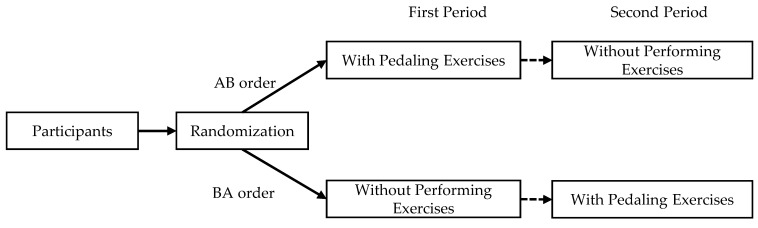
Crossover study design.

An announcement invitation was issued and distributed in King Saud University in order to recruit adult participants. In the annunciation, the participants were invited to participate in two assembly tasks at different time periods. Once the responses were received, participants were scheduled. The two tasks (e.g., performing pedaling exercises and without performing the exercises) were provided to participants at different time periods, and the order of tasks was randomized for each participant. Participants were randomly allocated to the AB or BA sequence (A and B referred to task A and task B) using a counterbalancing method. Participants in the AB sequence received task A (with performing pedaling exercises) in the first period, and after three months they received task B (without performing the exercises) in the second period. The remaining participants assigned to the BA sequence received task B first (assembling a keyboard while performing without pedaling) and then task A (with pedaling) (Figure 3).

In other words, half of the participants started the assembly task with cycling, whereas the other half started the assembly task without cycling. The first task was performed when the participant went to the experimental area for the first time, whereas the second task was executed three months after to avoid any learning effects. The health status of the participants did not change throughout the study. For each participant, the two assembly tasks were executed, these being performed while cycling at a comfortable speed and without cycling.

Following the electrode placement on the participant, the EEG and ECG data were recorded for 5 min while the participant was in rest (pre-test). Following that, the experiment was initiated. A stopwatch was used to record the time taken from the start of the keyboard assembly to its completion. Throughout the experiment, signals were measured and re-recorded for another 5 min while the participant was in rest (post-test). Following that, the individual completed the NASA TLX rating scale and a questionnaire about body discomfort.

### 2.5. Response Measures

#### 2.5.1. Performance Measures

The participants’ performances were evaluated using the number of errors made during task execution and the time required to complete the task. The keyboard assembly error was characterized as either incorrect button insertion or button insertion in the opposite direction. The completion time was the time required for each participant to complete an assembly task (keyboard assembly).

#### 2.5.2. Subjective Workload Ratings

The workload score was obtained from each participant by using the NASA TLX load index [59]. The NASA TLX involves six subscales, namely physical demand, mental demand, performance, temporal demand, effort, and frustration level.

#### 2.5.3. Subjective Body Discomfort Ratings

After completing the assembly activity, the discomfort survey form from the Vyas study [60] was used. The participants were asked to rate their level of discomfort in 12 different bodily parts (neck, shoulders, elbows, upper back, lower back, forearms, wrist/hands, hips, thighs, knees, legs, and ankles/feet) on a scale from 0 to 10 (where 0 indicated no discomfort and 10 denoted severe discomfort).

#### 2.5.4. Electroencephalography (EEG) Signal Responses

In this study, the EEG signals from the frontal regions (F3 or F4) were recorded using a high-quality, two-channel digital EEG preamplifier connected to ME 6000 (Mega Electronics Ltd., Kuopio, Finland). The validity and reliability of the instruments have been previously established (Ramadan and Alhaag [36]). The positions of the frontal region (F3 and F4) were determined in accordance with the 10–20 international standards for EEG electrode placement [61]. Subsequently, the Ag/AgCl disk electrodes, held using the emotive headset, were placed on definitively arranged regions (frontal region, forehead, and mastoid) after filling with super gel. The reference electrode was positioned above the mastoid region (behind the participant’s right ear), whereas the ground electrode was positioned on the participant’s forehead [36].

Mega Win 3.1 (Mega Electronics Ltd., Kuopio, Finland) was used to amplify and record EEG data at a sampling rate of 1000 Hz. The EEG signal was preprocessed to remove undesired signals (noise) using a low-pass filter composed of a four-pole elliptic filter (with a cutoff frequency of 32 Hz) to eliminate power-line and high-frequency noise. The filtered data set included all EEG waves (delta, theta, alpha, and beta). The EEG waves were extracted using the multilevel discrete wavelet transform (DWT), which incorporated the sub-band of the signal. A DWT with four levels based on Debauches 4 was used. Finally, the fast Fourier transform was used to determine the frequency contained in each DWT level.

The EEG indices, categorized into two sets, basic and ratio, were computed to contradict each other and amplify the differences. The response variables related to the EEG that were chosen as measures of mental workload were the EEG power of θ, α, β, θ/α, β/α, and (α + θ)/β indices. The fundamental indicators corresponded to the relative powers of θ, α, and β EEG bands. The equations of the relative powers for the bands are represented as follows in the order of appearance:

Relative power of θ = (power of θ)/(power of α + power of β + power of θ).

Relative power of α = (power of α)/(power of α + power of β + power of θ).

Relative power of β = (power of β)/(power of α + power of β + power of θ).

#### 2.5.5. Electrocardiographic (ECG) Response Analysis

At a sample rate of 1000 Hz, the ECG signals were continuously recorded for 5 min before the assembly task (pre-test session) and 5 min after the task was completed (post-test session). The ECG electrodes were placed on the right wrist, upper-right forearm (distal to the elbow), and upper-left forearm (distal to the elbow). The one-channel ECG preamplifier was connected to channels 2 and 3 of the ME 6000. Subsequently, the Kubios HRV software 2.2 was used for the analysis of the ECG signals [62].

The recorded ECG signal was pre-treated to eliminate unwanted signals (noise) by excluding the RR intervals that differed by more than 25% between two successive RR intervals [63]. Thereafter, traditional interpolation was employed to replace the measured RR intervals to ensure that the length of the data remained unchanged (i.e., the number of pulses remained constant). In addition, the prior smoothness approach with a lambda value of 1000 was applied to remove disturbing low-frequency baseline trend components [47].

HRV was used as a measure for mental workload in this investigation. The time and frequency domain indices were utilized to analyze the HRV. The following HRV variables were assessed in the time domain: the average of RR intervals (RR), average heart rate (HR), the standard deviation of normal RR intervals (SDNN), root mean square of successive differences in RR intervals (RMSSD), successive RR interval differences (NN50), and the percentage rate of successive RR intervals (pNN50). NN50 is the number of successive intervals with a difference greater than 50 milliseconds. Meanwhile, pNN50 is the percentage rate of a subsequent RR interval greater than 50 ms longer than the preceding interval.

Additionally, the frequency domain HRV parameters VLF, LF, and TP were evaluated. All components of the frequency domain were evaluated in absolute units (ms^2^). The ratio of the absolute power in the LF bands to the absolute power in the HF bands indicates the sign of the sympathetic–parasympathetic balance [62].

### 2.6. Statistical Analysis

A two-way repeated measure (ANOVA) was applied to test the main and interaction effects of assembly methods and session testing on heart rate variability and EEG power indices. The paired *t*-test was used to test the effect of assembly methods on completion time, percentage error, NASA TLX rating scale, and body discomfort rating scores. Design assumptions (normality, homogeneity of variance, and continuity of data) were examined to assure the reliability of the statistical analysis results. All data were normally distributed (preliminary assessment by Kolmogorov–Smirnov statistic tests with a Lilliefors correction) and the assumption of sphericity was met (preliminary assessment by Mauchly’s test). Descriptive statistics (mean and standard deviation) of all dependent variables were computed. In addition, the effect size was calculated based on the partial eta-squared value (ƞ^2^) to indicate the variance percentage in the dependent variables attributable to the particular independent variable. SPSS Version 23 (Armonk, NY: IBM Corp) was used to perform the statistical analysis.

## 3. Results

### 3.1. Completion Time and Percentage Error Analysis

The statistical analysis revealed no statistically significant difference in completion time between assembly methods (with or without pedaling exercise) (F (1, 15) = 4.187, *p* = 0.059). This finding indicated that pedaling during an assembly activity did not affect the participants’ performance in terms of task completion time. The mean times (standard deviation) of the executed assembly task while cycling and while not doing exercise were 20.171 (3.408) and 19.103 (2.339) min, respectively.

The error of the keyboard assembly was defined as either wrong insertion of the button to the keyboard or insertion of the button in the opposite direction and was counted after the keyboard assembly. The statistical analysis indicated no significant difference in the mean value of the percentage error between the assembly methods (F (1, 15) = 0.031, *p* = 0.863). This result indicated no significant difference in performing an assembly task while cycling on a bicycle or not on error numbers or error percentage.

### 3.2. Subjective Workload Analysis

The NASA TLX average score was computed using Sharek’s NASA-TLX Online Tool (Version 0.06) [64]. Statistical analysis revealed no statistically significant difference in NASA TLX scores between assembly methods (F (1, 15) = 2.300, *p* = 0.15). Additionally, the results demonstrated a statistically significant difference in physical effort scores between assembly methods (F (1, 15) = 9.359, *p* = 0.008). When assembling the keyboard while executing the pedaling exercise, the average of the physical workload ratings increased. This finding revealed that conducting the assembly process while pedaling increased the physical workload.

### 3.3. Body Discomfort Rating Analysis

The discomfort survey questionnaire of the Vyas study [60] was employed in this investigation in which participants rated their level of discomfort in 12 different bodily areas (e.g., neck, shoulders, elbows, upper back, lower back, forearms, wrist/hands, hips, thighs, knees, legs, and ankles/feet) on a scale from 0 to 10 (where 0 indicated no discomfort and 10 denoted severe discomfort). The average scores of 12 body parts were used for the analysis. The results demonstrated a statistically negligible difference in the average value of the assembly groups’ bodily discomfort scores (F (1, 15) = 0.032, *p* = 0.861).

### 3.4. ECG Response Analysis

Table 2 summarizes the mean (standard deviation) and the statistic values (*p*-value and partial eta-squared value (ƞ^2^) of the HRV parameters in time and frequency domains for the two sessions and for both methods. The effect size was computed using the partial eta-squared value (ƞ^2^) to represent the percentage of variance in dependent variables attributed to a given independent variable. HR, RR, SDRR, RMSSD, NN50, and pNN50 are the time domain values of the HRV that were utilized to compare the differences between the two assembly methods. Significant variations between the two approaches and the two sessions’ durations could be detected (*p* < 0.05). The results indicated that the session duration and assembly method significantly affected the mean RR interval (F (1, 15) = 14.78, *p* < 0.002, ƞ^2^ = 0.496 and F (1, 15) = 6.839, *p* < 0.019, ƞ^2^= 0.313, respectively).

In addition, the session time and assembly method had a significant effect on mean HR (F (1, 15) = 14.504, *p* < 0.002, ƞ^2^= 0.492 and F (1, 15) = 6.641, *p* < 0.021, ƞ^2^ = 0.492, respectively). Meanwhile, the interaction between the session time and assembly method had a significant effect on the mean HR, that is, F (1, 15) = 6.211, *p* < 0.025, ƞ^2^ = 0.293, as illustrated in Table 2. Moreover, the results indicated that neither of the assembly methods presented significant changes in the SDRR, RMSSD, NN50, pNN50, nor frequency domain measures.

**Figure 4:**
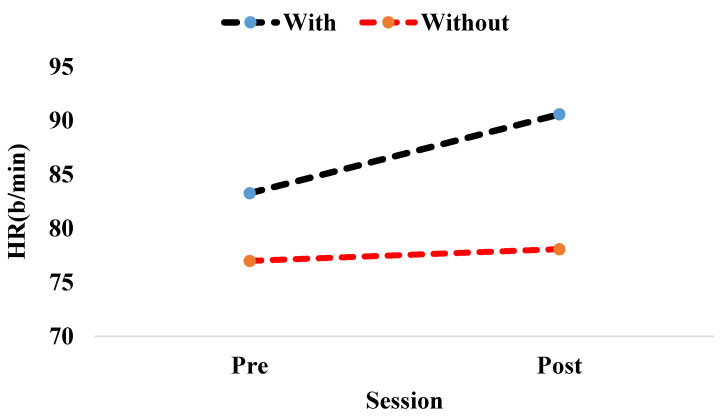
Interaction effects between the session and the assembly methods (with pedaling exercise and without pedaling exercise) on the HR.

The mean heart rate increased after participants completed the assembly task while executing the pedaling exercise; however, the mean heart rate remained relatively constant for participants who completed the assembly task without conducting the pedaling exercise, as illustrated in Figure 4. The aforementioned increase may have been caused by the physical effort resulting from the pedaling exercise.

### 3.5. EEG Power Spectra

Table 3 summarizes the mean (standard deviation) and the statistic values (*p*-value and partial eta-squared value (ƞ^2^) of the EEG indices of the times and assembly methods of the two sessions. The EEG power was measured at sites F3 and F4 (frontal lobe) and was analyzed by utilizing the repeated measure ANOVA with the within-variable “sessions”, including before performing the assembly task (pre) and after completing the assembly task (post). Moreover, the “assembly method” included conducting the pedaling exercise while assembling the keyboard and executing the assembly work without cycling exercise. The EEG indices were separated into two sets: basic (relative powers of θ, α, and β, ranging from 4 to 30 Hz) and ratio. The ratio indices θ/α, β/α, and (α + θ)/β were studied as well. The 1-min EEG data segments at the pre-and post-session were selected for the study.

As shown in Table 3, there was no significant effect of the assembly method on the relative power of the EEG for the theta, alpha, and beta rhythms, as well as for the θ/α, β/α, and (α + θ)/β logarithm. Only the session duration had a significant effect on the EEG’s alpha and beta power, the data suggested. Regardless of the assembling process, the beta and alpha (α) rhythms exhibited increasing patterns. Additionally, as indicated in Table 3, the interaction between the session’s duration and the assembly task did not indicate any significance for any of the EEG indices.

## 4. Discussion

In this study, 16 students from a university executed the keyboard assembly task. The effect of introducing the bicycle pedaling exercise on human workload and performance during the assembly activity was investigated using various measurements. Recently, several studies have suggested a moderate-intensity session of exercise for 20 min a day that will help improve a person’s health [65,66,67]. According to statistical analysis of the completion time, error percentage, participant self-reports, and the ECG and EEG signals, the keyboard assembly task performed concurrently with the pedaling exercise could not create mental workload or impair participant performance. The mental workload was assumed to be associated with the alertness and extension of mental information processing. However, we observed that the average completion time, error percentage, and average score for the NASA TLX were not significantly different between the approaches in this investigation.

No evidence of the effect of introducing pedaling exercises while executing an assembly activity on human performance was observed based on the average completion time and error percentage. This study demonstrated that performance levels were stable and that all participants were capable of completing the same task in approximately the same amount of time and with the same number of errors. This study suggests that the tasks may be quite straightforward. Additionally, no variation in the NASA TLX score was seen between assembly tasks performed with and without pedaling activities. However, the average scores of the physical workload of the NASA TLX increased by 42.5% for the case of assembling the keyboard while performing pedaling exercises. The study’s finding revealed that executing the assembly task while pedaling resulted in increased physical workload, which may be attributed to the exercise. Previous studies have revealed varied results at a work station wherein workers were engaged with a treadmill [68,69,70,71], pedaling [72], and other innovative styles [73] compared with the standard seating for various computer processes (i.e., precision, time of typing, word amount). The results of this study were consistent with those of Elmer and Martin [74] that indicated the pedaling workstation as an additional practical option to increase the physical activity of workers in the office without compromising their typing performance.

For years, it was known that the variation in brain alertness included several differences in oscillatory brain activities, and the effect of attention level could be reflected in the EEG. Currently, EEG is a significant and reliable predictor of mental fatigue [33,36]. Numerous studies have investigated the variations in EEG waves during continuous and demanding tasks to increase the EEG’s theta power on the frontal cortex. This increase was described in the tasks that included visual research [35], presentation of 3D display [36], and loading of working memory [33]. Additionally, all difficult and cognitive tasks resulted in a drop in the EEG’s alpha power. This decrease occurred in the frontocentral and parietal regions [37,38]. Zhao et al. [39] found a large rise in the EEG’s alpha and theta powers and a significant drop in the beta power in several scalp areas. In addition, they reported that the beta power decreased in the frontal regions. Furthermore, they discovered that the beta rhythm was drastically reduced in the frontal areas. The results of this investigation demonstrated that there were no significant changes between the assembly methods in terms of the EEG’s relative power regarding the theta, alpha, and beta rhythms as well as θ/α, β/α, and (α + θ)/β logarithms. In addition, the interaction between the sessions and assembly groups was insignificant for all EEG indices. The EEG response revealed that no procedure for achieving the mental burden was detectable. This finding could be because the task duration was insufficient for participants to achieve a particular level of fatigue or because participants had significant typing experience. Additionally, no equivalent previous research has been conducted in which EEG data were taken during cycling exercises.

Significant variations in HR and RR were identified among participants who completed the assembly task while performing the pedaling exercise and those who completed the assembly task without performing the pedaling exercise (*p* < 0.05), as assessed by HRV. We determined that the mean HR of the participants increased by 10.9% after they had executed the assembly task while performing pedaling exercises. Meanwhile, the mean RR interval decreased by 10.9% after the participants had executed the assembly task while performing pedaling exercises. This decrease and increase in values may have been caused by the physical effort resulting from the pedaling exercises. The study’s findings support the findings of Elmer and Martin [74] and Straker et al. [72]. Straker et al. [72] reported that the HR values increased by 25% when the participants were pedaling and typing simultaneously compared with the HR values obtained from normal sitting and typing. This significant difference may have occurred because of the pedaling speed. Moreover, the results of this investigation demonstrated that neither assembly approach significantly altered SDRR, RMSSD, NN50, pNN50, nor frequency domain measurements.

## 5. Conclusions

The main contribution of this study was to introduce and interpret the EEG and HRV parameters while the participant performed physical work associated with a cognitive task. This study concluded that participant performance was unaffected by performing a pedaling exercise while doing the assembly activity. Thus, administrations should encourage their employees to perform exercise in short sessions (at least 20 min) of moderate-intensity exercise by ergonomic cycling throughout the workday in order to improve a person’s physical health without interfering with the effectiveness of work. Further, the participants pedaled at a comfortable speed with light power output for continuous pedaling without becoming exhausted.

### Limitations

Despite the authors’ repeated efforts to recruit females through classified flyers and pamphlets in the girls’ section, only male participants responded and were recruited for the study. Another limitation exists regarding the participants assigned to this study. The participants were chosen from a university’s student body, and none had prior assembly experience. The results of this study can be applied to males but a generalization of the current study findings to females and different workers may be difficult. Other power outputs of ergonomic bikes should be evaluated to generalize the findings and to determine the best power outputs that should be employed in work environments.

## Figures and Tables

**Table 1:** Detailed information on the participants.

Measure	Mean	(Std.)	Measure	Mean	(Std.)
Age (yrs.)	30.19	2.60	Elbow Height (cm)	107.48	3.80
Weight (kg)	74.43	14.87	Body Mass Index (kg/m^2^)	26.06	4.62
Stature Height (cm)	168.84	4.72	Elbow Sitting Height (cm)	33.55	2.20

**Table 2:** Mean (standard deviation) values of the HRV before and after assembly task with and without pedaling.

Parameters	Mean (SD)				Statistics P (ƞ^2^)		
Session	Pre		Post		Session (ƞ^2^)	Assembly Methods (ƞ^2^)	Interaction (ƞ^2^)
Assembly Methods	With	Without	With	Without			
mRR (ms)	735.77 (103.1)	797.14 (114.48)	680.04 (108.98)	787.48 (118.85)	0.002 (0.496) *	0.019 (0.313) *	0.063 (0.212)
STDRR (ms)	28.76 (14.8)	27.53 (12.69)	28.61 (12.21)	29.41 (15.57)	0.600 (0.019)	0.952 (00)	0.614 (0.015)
mHR (bpm)	83.28 (11.08)	77.00 (12.06)	90.59 (13.48)	78.09 (12.38)	0.002 (0.492) *	0.021 (0.307) *	0.025 (0.293) **
RMSSD (ms)	23.56 (16.54)	24.34 (14.26)	21.9 (12.52)	25.79 (15.23)	0.946 (0.00)	0.585 (0.020)	0.377 (0.052)
NN50	6.81 (10.36)	5.25 (7.77)	5.00 (6.98)	5.94 (7.22)	0.623 (0.017)	0.901 (0.001)	0.279 (0.078)
PNN50 (%)	7.94 (13.87)	8.16 (12.91)	6.58 (10.44)	9.02 (12.11)	0.845 (0.003)	0.734 (0.008)	0.349 (0.059)
VLF (ms^2^)	109.98 (144.52)	110.36 (112.54)	116.08 (176.71)	54.82 (50.79)	0.27 (0.08)	0.476 (0.034)	0.113 (0.159)
LF (ms^2^)	520.44 (570.34)	378.5 (291.85)	619.87 (747.42)	635.94 (1132.63)	0.275 (0.079)	0.721 (0.009)	0.652 (0.014)
HF (ms^2^)	295.52 (461.16)	335.29 (446.11)	211.47 (263.48)	399.06 (559.65)	0.849 (0.002)	0.43 (0.042)	0.072 (0.2)
LF/HF (ms^2^)	4.53 (3.98)	3.49 (4.15)	7.8 (10.4)	3.64 (4.44)	0.119 (0.154)	0.159 (0.128)	0.304 (0.070)

P * = the significance between pre and post of the task; P ** = the significance interaction between type of task and test time; mRR = average of RR intervals; STDRR = standard deviation of normal RR intervals; mHR = mean heart rate; RMSSD = root mean square of successive differences in RR intervals; NN50 = successive RR interval differences; PNN50 = percentage rate of a successive RR intervals; VLF: very low frequency; LF: low frequency; HF: high frequency.

**Table 3:** Mean (standard deviation) values of relative power of EEG for the theta, alpha, beta rhythms and θ/α, β/α, and (α + θ)/β logarithms before and after assembly task with and without pedaling.

Parameters	Mean (SD)				Statistics P (ƞ^2^)		
Session	Pre		Post		2	Assembly Methods (ƞ^2^)	Interaction (ƞ^2^)
Assembly Methods	With	Without	With	Without			
Theta (θ)	321.41 (216.39)	298.28 (127.3)	956.39 (2425.87)	543.92 (1019.19)	0.19 (0.112)	0.539 (0.026)	0.571 (0.022)
Alpha (α)	568.4 (409.76)	598.8 (523.95)	891.66 (765.92)	760.08 (628.17)	0.039 (0.255) *	0.689 (0.011)	0.522 (0.028)
Beta (β)	333.94 (147.7)	310.57 (129.93)	556.33 (560.06)	420.07 (274.81)	0.043 (0.246) *	0.389 (0.05)	0.481 (0.034)
θ/α	0.77 (0.45)	0.82 (0.5)	0.74 (0.68)	0.84 (0.62)	0.943 (0.00)	0.579 (0.021)	0.752 (0.007)
β/α	0.81 (0.42)	0.88 (0.61)	0.76 (0.40)	0.96 (0.92)	0.874 (0.002)	0.282 (0.077)	0.265 (0.082)
(α + θ)/β	2.77 (1.50)	2.86 (1.53)	2.88 (1.75)	2.82 (1.4)	0.898 (0.001)	0.945 (0.00)	0.772 (.087)

P * = the significance between pre and post of the task.

## Data Availability

All data are included in this paper.
